# Interplay of Interleukin-1**β** and Curcumin on VEGF Expression in Breast Cancer Cells

**DOI:** 10.32604/or.2025.072793

**Published:** 2026-02-24

**Authors:** Norbert Nass, Atanas Ignatov, Thomas Kalinski

**Affiliations:** 1 Institute of Pathology, University Hospital Brandenburg/Havel, Brandenburg Medical School Theodor Fontane (MHB), Brandenburg an der Havel, 14770, Germany; 2 Institute of Pathology, Otto von Guericke University Magdeburg, Magdeburg, 39120, Germany; 3 Department of Obstetrics and Gynecology, Otto von Guericke University Magdeburg, Magdeburg, 39108, Germany

**Keywords:** Breast cancer, curcumin, interleukin-1β, p38-MAPK, ERK, VEGF

## Abstract

**Objectives:**

Vascular endothelial growth factor (VEGF) regulates tumor vascularization in response to hypoxia and inflammatory signals. The polyphenol curcumin is supposed to interfere with inflammation-induced VEGF secretion and might therefore support anti-VEGF-based treatments. We aimed to investigate the interaction between curcumin and the inflammatory cytokine Interleukin-1β (IL-1β) for VEGF secretion in breast cancer cell lines representing major breast cancer subtypes.

**Methods:**

VEGF in cell cultures was detected by Western blot and enzyme-linked immunosorbent assay (ELISA). Kinase phosphorylation was investigated by Western blotting. Gene expressions were analyzed by correlation tests. VEGF was evaluated in a retrospective breast cancer cohort by immunohistochemistry. Survival analysis was performed by the Kaplan-Meier algorithm.

**Results:**

VEGF secretion and kinase signaling in response to IL-1β and curcumin varied significantly for the cell lines MCF-7 (Luminal A), SK-BR-3 (HER2/neu+), MDA-MB-231, and UACC-3199 (triple negative breast cancer). All cell lines increased VEGF secretion under hypoxia, but IL-1β increased VEGF secretion only in MCF-7 cells. Curcumin inhibited VEGF secretion in MDA-MB-231, but increased it in MCF-7 and UACC-3199 cells. Curcumin induced phosphorylation of extracellular signal-regulated kinase (ERK) and p38-mitogen-activated protein kinase (p38-MAPK). However, inhibitor experiments demonstrated that ERK was more important for VEGF secretion. In gene expression data from the METABRIC study, no clear correlation of hypoxia-induced factor (HIF1A), IL-1β, and VEGF mRNA expression was observed; however, a suggested crosstalk of hypoxia and inflammatory pathways was observed.

**Conclusion:**

These dissimilar responses of breast cancer cell lines suggest that therapy efficiency with anti-VEGF, anti-IL-1β, or curcumin will also vary within breast cancers.

## 1 Introduction

Breast cancer (BC) is the most frequent cancer in women, with about 2.2 million new cases (in 2020) worldwide. This heterogeneous neoplasm is still the leading cause of cancer-related mortality in women [1]. Pathologically, BC is classified by histology first. The major, but highly heterogeneous BC class is the “invasive carcinoma of no special type”, which is defined by not showing sufficient characteristics to be allocated to a specific histological type. Major histologically defined BC types are the lobular, cribriform, mucous, medullary, squamous, and papillary carcinomas. Despite the growing importance of molecular pathology for BC, determining the morphological subtypes is still valuable, as these subtypes are strongly associated with distinct prognostic outcomes [2]. Four biomarkers are further required for defining the most important molecular BC subtypes in a clinical setting. Here, BC is classified according to the expression of receptors for estrogen (ER), progesterone (PgR), epidermal growth factor receptor-2 (HER2/neu, erbb2), and the proliferation marker Ki67. These biomarkers define the intrinsic molecular subtypes [3] of breast cancer without extensive gene expression analysis in a clinical setting [4]. The molecular subtypes of BC are the luminal A subtype, expressing the ER and/or PgR without HER2/neu-overexpression and a low percentage of Ki67-positive, proliferating cells. Luminal B also expresses the ER/PgR receptors and is further characterized by either high Ki67 frequency or HER2/neu-overexpression [5]. The next subtype, overexpressing HER2/neu in the absence of ER/PgR, is called HER2/neu-positive (non-luminal). Finally, the absence of these three receptors defines triple-negative breast cancer (TNBC), which closely resembles the molecularly defined basal subtype [6].

Luminal tumors represent about two-thirds of BCs. Interestingly, PgR is mostly expressed in ER-positive tumors, but rare PgR-positive but ER-negative cases have also been described [7]. Patients with ER-positive tumors have a far better prognosis than the other subtypes and can be treated by endocrine therapy, either with selective estrogen mediators such as tamoxifen for premenopausal women or aromatase inhibitors for postmenopausal patients [8]. Luminal B cases are a more aggressive form of luminal breast cancer, and therefore frequently treated by additional chemo- and/or radiotherapy or an HER2/neu targeted therapy. The decision to add a potentially harmful chemotherapy can be difficult. Thus, several gene expression tests have been developed, assessing the tumor recurrence rate much better than applying hormone receptor expression and Ki67 alone [9,10]. For HER2/neu-overexpressing cases, a targeted therapy using monoclonal anti-HER2/neu-antibodies (e.g., Trastuzumab) was approved by the FDA in 1998. This therapy has greatly improved the outcome of this subtype and improved antibody-drug conjugates, even for the treatment of low and ultra-low HER2/neu expressing cases are now available [11]. However, no targeted therapy for the remaining heterogeneous group [12] of TNBC patients (10%–20%) is currently available; thus, chemo- and radiotherapy are typically applied. This type of cancer also has the worst outcome [13]. Nevertheless, the introduction of immunotherapy using checkpoint inhibitors provided an additional treatment option [14]. A subgroup of BC is caused by germ-line mutations, most commonly in BRCA1 and BRCA2 (about 5% of BC cases), and these often exhibit a TNBC-like histological phenotype [15].

Vascular endothelial growth factor (VEGF) is critical for tumor progression as it drives angiogenesis, which is critical for nutrient and oxygen supply, particularly for larger tumors [16]. VEGF expression is regulated by at least two major pathways. Firstly, its expression is triggered by hypoxia, mediated by hypoxia-induced factor 1α (HIF1A) [17]. Secondly, it responds to inflammatory signals, such as Interleukin-1β (IL-1β). This cytokine participates in malignant and inflammatory processes [18] and, among other functions, regulates the expression of VEGF in several cancer entities via the activation of the transcription factor nuclear factor kappa-light-chain-enhancer of activated B cells (NF-κB) [19,20]. IL-1β is therefore considered a negative prognostic factor, especially due to its role in driving metastasis [21].

Plant-derived compounds have long been used in traditional medicine. A rising number of substances have been isolated and tested for therapeutic efficacy, especially in cancer treatment. These compounds belong to various chemical classes, with phenolic plant secondary metabolites being most extensively studied (for review see: [22]). One of the most extensively studied compounds is curcumin. It is the major constituent of turmeric and curry and is responsible for the characteristic yellow color [23]. It has been attributed a magnitude of beneficial effects, ranging from anti-cancer, anti-oxidative effects to plasma cholesterol control [24,25].

Molecularly, curcumin inhibits IL-1β-receptor signaling by preventing the recruitment of interleukin-associated kinase (IRAK) and is therefore anti-inflammatory [26]. Nevertheless, a multitude of other targets have been described [27]. In chondrosarcoma [19] and fibrosarcoma [28] cell lines, curcumin downregulates VEGF and other NF-κB-regulated genes at concentrations that inhibit the activity of this transcription factor. In breast cancer models, curcumin exerted a similar suppression of VEGF expression in the triple-negative MDA-MB-231- [29] as well as in the estrogen receptor-positive T-47D cell line [30].

We therefore hypothesized that an inhibition of the inflammatory transcription factor NF-κB by curcumin would counteract the IL-1β-mediated increase in VEGF, thereby enhancing anti-VEGF antibody treatment effectiveness. For BC, the relevance of IL-1β signaling for VEGF secretion, as well as the impact of curcumin on BC subtypes still remains not sufficiently understood. A deeper understanding of the responses of the different BC subtypes or even individual tumors to IL-1β and curcumin with respect to tumor vascularization would be crucial for guiding therapy.

In this study, we investigated the interaction of IL-1β and curcumin on VEGF secretion in cell lines representing the major BC subtypes. Furthermore, we correlated VEGF expression with BC subtypes and outcome in a retrospective BC cohort by immunohistochemistry (IHC). Finally, to further assess the importance of this signaling cascade, we explored the METABRIC breast cancer dataset [31] and the Cancer Cell Line Encyclopedia [32] for expression of IL-1β, HIF1A, and VEGFA. Based on these data, we intended to estimate the potential effectiveness of combining anti-IL-1β/-VEGF directed therapy, i.e., with curcumin, across the BC subtypes. We propose that such data will become the basis for personalized therapy strategies in the future.

## 2 Materials and Methods

### 2.1 Cells and Cell Culture

All cell lines MCF-7 (ATCC-HTB-22), SK-BR-3 (ATCC-HTB-30), MDA-MB-231 (ATCC-HTB-26), and UACC-3199 (ATCC-CRL-2983) were obtained from the American Type Culture Collection (ATCC, via LGC Standards, Wesel, Germany). Cells were authenticated by short tandem repeat (STR) analysis and tested to be free of mycoplasma contamination. Cells were maintained at 37°C with 5% CO_2_-enriched atmosphere in RPMI-1640 medium without phenol-red (#FG1215, Biochrom, Berlin, Germany) but supplemented with 9% fetal calf serum (FCS, #0299G, Biochrom) and glutamax (#35050-038, Gibco, Thermo Fisher Scientific, Waltham, MA, USA) as recommended. Cells were provided with fresh medium every two to three days and subcultured weekly before reaching confluence by using trypsin/EDTA (#L2153, Biochrom). Passage number of all cell lines was limited to 50.

### 2.2 Cell Stimulation and Viability Assay

For VEGF determination by enzyme-linked immunosorbent assay (ELISA), 8 × 10^4^ cells per well were seeded in a 24-well plate (Greiner Bio-One, Frickenhausen, Germany). For viability tests, 4 × 10^4^ cells per well were seeded into 48-well plates (Greiner Bio-One) in serum-supplemented RPMI-1640 medium. This corresponded to a confluence of about 50%. After 24 h, the medium was removed and replaced with fresh FCS-supplemented medium, containing the compound under study (UO126, SB203580, and curcumin) or solvent (DMSO, 0.1%). Kinase inhibitors UO126 (1 µM, #19-147, Merck Millipore, Darmstadt, Germany) and SB203580 (1 µM) (S1076, Selleckchem, Houston, TX, USA via VWR-International, Darmstadt, Germany) as well as curcumin (#C1386, Sigma-Aldrich, St. Louis, MO, USA) and solvent DMSO were added (1/1000), 2.5 h before adding IL-1β (#200-01B, PeproTech, Thermofisher, Darmstadt, Germany). This ensured their entry into the cells. Cell culture supernatant was sampled after 24 h for VEGF determination.

For VEGF determination by Western blot, cells were incubated with IL-1β for up to 24 h in 5 mL serum-free medium in T75 cell culture flasks (Greiner Bio-One) at 50% confluence. After 1, 3, 6, 12, and 24 h incubation, the culture supernatant was sampled for VEGF-determination and concentrated approximately 50-fold using Amicon ultracentrifugal filters (#UFC9003, Millipore) by centrifugation at 4000× *g* for up to 60 min at 4°C [19].

For hypoxia experiments, 8 × 10^4^ cells per well were seeded into 24-well plates in full medium. The next day, medium was replaced with fresh full medium, and the plate was placed into a hypoxic bag (#1138290001, Anaerocult A mini, Merck-Millipore), sealed with an anaeroclip (#114226, Merck-Millipore), and controlled for anaerobic conditions using anaerotest (#132371, Merck-Millipore) for 24 h. A control plate was kept under normoxic conditions. Cell culture supernatant was sampled after 24 h for VEGF determination, and cells for RNA isolation.

For viability assays, cells were incubated for three days in full medium containing curcumin at the indicated concentrations. Afterwards, the medium was removed, and PBS containing resazurin (10 µg/mL, #A2830, Applichem, Darmstadt, Germany) was added. Cells were incubated with resazurin for 1 to 3 h until a color change became visible. Fluorescence was determined in a Glomax multidetection plate reader (#E7031, Promega, Mannheim, Germany) at wavelengths 525/580–640 nm (excitation/emission, fluorescence module “green”) as previously described [33]. Fluorescence of control cells was set to 1.

To determine early signaling events, 8 × 10^4^ cells were seeded into each well of a 24-well plate, grown to confluence, and then serum-starved for 24 h. IL-1β (10 ng/mL) was added for ten minutes before the medium was aspirated and the cells lysed with 50 µL Tris–HCl buffer 50 mM, pH 6.8 containing SDS (2%) and phosphatase and protease inhibitors (Sigma-Aldrich).

### 2.3 Western Blotting

Denatured and reduced proteins were separated on denaturing polyacrylamide gels (15% for VEGF and 12% for all other applications). Transfer to nitrocellulose membranes (Millipore) was performed by semi-dry blotting in CAPS buffer (50 mM, pH 10, Sigma-Aldrich) supplemented with methanol (10%) and 3-mercaptopropionic acid (1 mM, Sigma-Aldrich) as described earlier [34]. Filters were blocked with TBS/NP40/BSA (Tris–HCl buffer 50 mM, pH 7.4, containing bovine serum albumin (BSA, fraction V, #0163.4, Carl-Roth, Karlsruhe, Germany) [2%]) Nonidet-P40 (NP-40, #1694, Applichem) [0.2%]) and sodium azide (0.03%). Primary antibodies (ERK #4695, p-ERK #3179, p38-MAPK #9212, p-p38-MAPK #4511, AKT #9272, p-AKT #4060, p65 #8242, p-p65 #3033, IκBα #4814, p-IκBα #9246 from Cell Signaling Technology, Frankfurt/Main, Germany, and β-actin, clone AC-15, #A5441, Sigma-Aldrich) were applied in the same buffer overnight at 4°C, diluted 1:1000 or 1:5000 (anti β-actin). Washing (3 × 15 min) and incubation with secondary antibody (1:10,000, 1 h) conjugated to horseradish peroxidase (goat-anti rabbit HRP #111-035-144 or goat anti mouse-HRP # 415-035-166, both Jackson-Immuno, Cambridgeshire, UK via Dianova, Hamburg, Germany) and three further washes (15 min) were performed at room temperature in TBS/NP40 with reduced BSA concentration (0.1%) before detection of the signals by enhanced chemiluminescence (# WBKL SO 100, Millipore) in a GeneGnome 5 (Syngene, Cambridge, UK) or INTAS Chemostar plus XL (Intas, Göttingen, Germany) CCD-camera system. Signals were compared to the signal of untreated control cells.

### 2.4 VEGF Determination

VEGF-A in the cell culture supernatants was determined by a commercial ELISA, according to the manufacturer’s recommendations (VEGF-DuoSet, #DY293B, R&D, Wiesbaden, Germany), and concentrations were calculated using the external standard provided. Depending on the expected VEGF levels, either 10 µL (MDA-MB-231) or 50 µL (MCF-7, SK-BR-3, UACC-3199) of the cell culture supernatant was analyzed by this assay. For comparing the VEGF concentrations between the cell lines, the VEGF concentration is presented. For estimating the effect of treatments, the relative VEGF concentrations to controls were calculated.

### 2.5 VEGF Determination by IHC in Clinical Samples

VEGF was stained in formaldehyde-fixed paraffin-embedded (FFPE) tissue of 230 breast cancer patients. This collective has been described earlier [35], and the study has been approved by the Ethics Committee of the Otto von Guericke University, Medical Faculty (AKZ 114/13, 2013) according to the Declaration of Helsinki. Informed consent was waived due to the sole use of residual material for diagnostic purposes, and contacting patients would have required disproportionate effort in accordance with the statement of the German Central Ethics Commission (Deutsches Ärzteblatt 100, 23, 2003). After deparaffinisation, epitopes were demasked with TRIS/EDTA buffer (pH 9) for 20 s at 125°C in a pressure cooker. Primary rabbit polyclonal antibody A20 (Antibody registry: AB_2212984, #sc-152, St. Cruz Biotechnology, Heidelberg, Germany) was applied in Ventana dilution buffer in a dilution of 1:50 for 32 min at 37°C in an automated slide staining system (Ventana Benchmark XT, Ventana, Mannheim, Germany). Detection was performed using the iView DAB staining reagents (# 06396500001, Ventana). The staining was assessed and discussed by TK and NN according to the four eyes principle. An immunoreactive score (IRS) was determined according to Remmele et al. [36] by assessing staining intensity (0 = no staining, 1 = weak staining, 2 = intermediate staining, 3 = strong staining) and percentage of positive tumor cells (1 ≤ 10%, 2 = 11%–50%, 3 = 51%–80%, 4 = > 80%) followed by multiplication of these two parameters. The IRS therefore ranges from 0 to 12. For cut-off determination, all possible IRS-cut-off values were used in a Kaplan-Meier analysis for relapse-free survival. VEGF “high” expression was defined for tumors with an IRS ≥ 6 based on these results. 6 is also the median of the IRS score.

### 2.6 Analysis of Public Gene Expression Databases

Gene expression data of the METARIC breast cancer study [31] were either analyzed with the tools provided at the cbioportal [37] website or downloaded (http://www.cbioportal.org/) and imported into SPSS (vers 24, IBM Corp., IBM, Armonk, NY, USA) and then used for cross-tabulation, correlation, and survival analysis. Euclidean cluster analysis was performed using the One Matrix CIM online tool at CIMminer (https://discover.nci.nih.gov/cimminer/home.do, accessed on 15th July 2025) [38].

### 2.7 RNA Extraction and Quantitative Reverse-Transcriptase Polymerase Chain Reaction (qRT-PCR)

RNA was extracted from cell cultures using the NucleoSpin RNA kit (#704933, Macherey-Nagel, Düren, Germany) according to the manufacturer’s recommendations. cDNA was synthesized using Script Reverse Transcriptase and oligo dT primers from 1 µg of total RNA (# PCR505 and # PM305, Jenabioscience, Jena, Germany). Real-time PCR was performed in a 20 µL volume using a Roche LightCycler 1.0 and the LightCycler® FastStart DNA Master SYBR Green I reagents (#03003230001, Roche-Life Science, Mannheim, Germany). Primers were used at 0.25 pmol/µL final concentration. For VEGFA, forward primer TGCATTGGAGCCTTGCCTTG and reverse primer CGGCTCACCGCCTCGGCTTG were used. For HIF1A, forward primer TGCTGGATCACAGACAGCTCA and reverse primer ACCACGTACTGCTGGCAAAGC were used. Normalization was done towards RPL13 (forward primer: CCTGGAGGAGAAGAGGAAAGAGA, reverse primer: TTGAGGACCTCTTGTGTATTTGTCAA). PCR conditions for both PCRs were annealing for 20 s at 60°C, followed by synthesis for 1 min at 72°C and 20 s of denaturation at 96°C. PCR was done for 45 cycles. All primers were obtained from Biomers (Ulm, Germany). Relative mRNA amounts were calculated using the 2^−ΔΔCt^ method [39].

### 2.8 Statistical Analysis

For statistical calculations, SPSS version 24 (IBM, Armonk, NY: IBM Corp. USA) was used. Means were compared by one-way ANOVA with post-hoc analysis by the Tamhane T2 or Games-Howell method, assuming unequal variances. When relative values were averaged, the geometrical mean was used. Data are given +/- standard error when not indicated otherwise in the legends of the figures or tables. For comparing inhibitory effects on the IL-1β-induced stimulation of VEGF secretion, this inhibition was calculated relative to the VEGF increase of the individual experiment. For correlation analysis of VEGF immunoreactive score with hormone receptor status, cross-tabulation analysis was performed with significance level *p* determined by Pearson’s R test (interval by interval). Crosstabulation analysis with Fisher’s exact test or Pearson chi-square was applied to show the correlation of categorical parameters.

## 3 Results

### 3.1 VEGF-Secretion by Mammary Carcinoma Cell Lines

**Figure 1:**
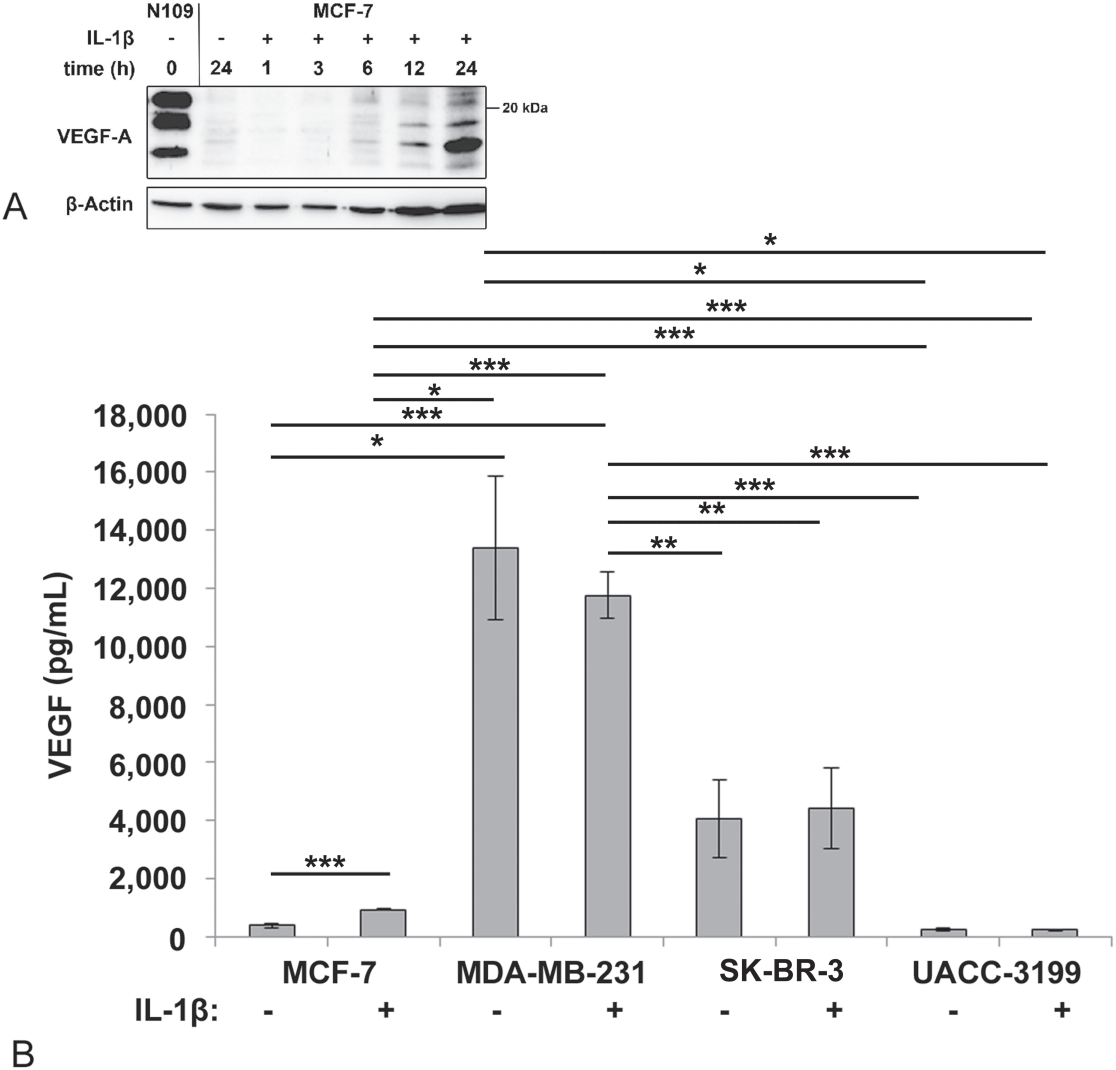
(**A**) Secretion of vascular endothelial growth factor (VEGF) in response to Interleukin-1β (IL-1β) by MCF-7 cells as demonstrated by Western blotting. Cells were inoculated in serum-free medium, and the medium was sampled, concentrated, and analyzed by Western blotting as described in the method section. N109 cell culture supernatant was applied as a positive control. (**B**) VEGF in the medium of breast cancer cells incubated for 24 h with and without IL-1β as determined by enzyme-linked immunosorbent assay (ELISA). The average of 4 independent experiments with at least 2 replicas each, and the standard error is shown. ***p < 0.001, **p < 0.01, *p < 0.05 (one-way ANOVA, Games-Howell post hoc test)

We first showed that MCF-7 cells secreted VEGF into the medium under serum-free conditions by Western blotting (Fig. 1A), as done for chondrosarcoma cell lines in our previous publication [19]. Under these conditions, MCF-7 cells do not proliferate further. We used a renal cell carcinoma cell line designated N109, known to secrete high amounts of VEGF, as a positive control for the procedure [40]. The observed pattern of VEGF isoforms was comparable to N109 as well as chondrosarcoma cell lines SW1353 and C3842 [19]. β-actin was detected as a positive control for the blotting process, although intact cells were not present in the concentrated growth media. Nevertheless, β-actin is always present in cell culture supernatants due to ongoing cell death, and therefore, a slight increase with incubation time was observed.

For further experiments, we detected VEGF by ELISA in serum-containing medium, as this method provides quantitative data, and curcumin was also less toxic when serum was present. The investigated breast cancer cell lines secreted significantly different amounts of VEGF (Fig. 1B). The triple negative cell line MDA-MB-231 produced the highest amounts of VEGF, followed by the ER-negative, but HER2/neu positive SK-BR-3, and the ER-positive MCF-7, and the ER-negative, but highly DNA-methylated UACC-3199 cell line. IL-1β treatment increased secretion of VEGF only in the MCF-7 cell line (Fig. 1B). To exclude potential effects of serum-derived signals, we repeated this experiment in serum-depleted medium. The results were consistent with MCF-7 showing a significant increase in extracellular VEGF upon IL-1β stimulation (Fig. S1).

### 3.2 VEGF-Expression in Tumor Tissue Samples

**Figure 2:**
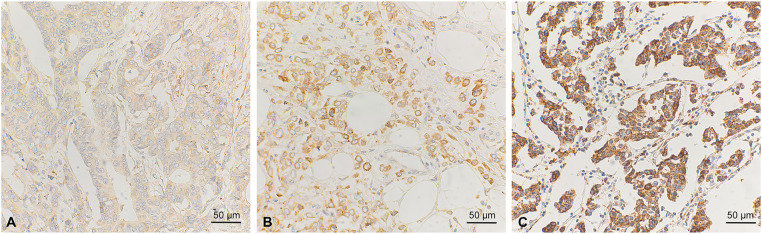
Immunohistochemical staining of VEGF in breast cancer samples. Weak (**A**), intermediate (**B**), and strong staining intensity (**C**) are shown. Scale bar corresponds to 50 µm

As the next step, we stained tumor specimens of our BC cohort [35,41,42] for VEGF-expression using IHC (Fig. 2). Positive staining was observed and scored in the cytoplasm of tumor cells. We correlated the VEGF-status using a VEGF-immunoreactive score (VEGF-IRS) ≥ 6 as a cut-off, with clinico-pathological parameters and patients’ survival (Table 1). VEGF was significantly higher in ductal, compared to lobular BC, but no other statistically significant association was found. For survival, we also found no significant correlation of VEGF-IRS (log rank *p* < 0.05), neither for the whole study population nor in subtype specific analysis for ductal, lobular, and TNBC. However, according to the kmplot webtool [43] (kmplot.com, accessed on 2 August 2025), VEGF-mRNA is a negative prognostic factor for disease-free survival (HR = 1.43, 95%CI: 1.29–2.59, *p* = 2.4 × 10^−11^) for all subtypes included (*n* = 4929).

**Table 1:** Clinico-pathological parameters and vascular endothelial growth factor (VEGF) status. An IRS ≥ 6 was considered as “VEGF-high” status

Parameter	Number	VEGF Low/High	VEGF High (%)	*p*-Value
All patients	230	122/108	47.0%	
Histology				
Ductal	175	85/90	51.4%	0.005
Lobular	43	32/11	25.6%	
Other	11	4/7	63.6%	
Menopausal−	45	25/20	44.4%	0.741
Menopausal+	185	97/88	47.6%	
T < 2	117	58/59	50.4%	0.294
T ≥ 2	113	64/49	43.3%	
Ki67 < 23	97	50/47	48.5%	0.306
Ki67 ≥ 23	52	22/30	57.7%	
N0	147	80/67	45.6%	0.581
N1	82	41/41	50.0%	
G1	27	14/13	48.1%	0.534^$^
G2	132	74/58	43.9%	
G3	71	34/37	52.1%	
ER−	46	21/25	54.3%	0.322
ER+	184	101/83	45.1%	
Luminal A	127	74/53	41.7%	0.201
Luminal B	57	27/30	52.6%	
PR−	101	50/51	50.5%	0.355
PR+	129	72/57	44.2%	
HER2−	182	98/ 84	46.2%	0.745
HER2+	48	24/24	50.0%	
No TNBC	198	109/89	44.9%	0.181
TNBC	32	13/19	59.4%	
No radiotherapy	72	49/23	31.9%	0.003
Radiotherapy	158	73/85	53.8%	
No chemotherapy	122	69/53	43.4%	0.290
Chemotherapy	108	53/55	50.9%	
No endocrine therapy	39	17/22	56.4%	
Tamoxifen	126	70/56	44.4%	0.424
Aromatase inhibitor	64	34/30	46.9%	
No tamoxifen relapse	80	43/37	46.3%	0.710
Tamoxifen relapse	46	27/19	41.3%	

Note: Total numbers may vary since not all parameters were available for every patient. For 2 × 2 contingency tables, Fisher’s exact *p* is provided; otherwise, significance was calculated using Pearson Chi-Square^$^. VEGF: vascular endothelial growth factor; KI67: proliferation-related Ki-67 antigen; PR: progesterone receptor, ER: estrogen receptor, HER2: human epidermal growth factor receptor 2, TNBC: triple negative breast cancer.

**Figure 3:**
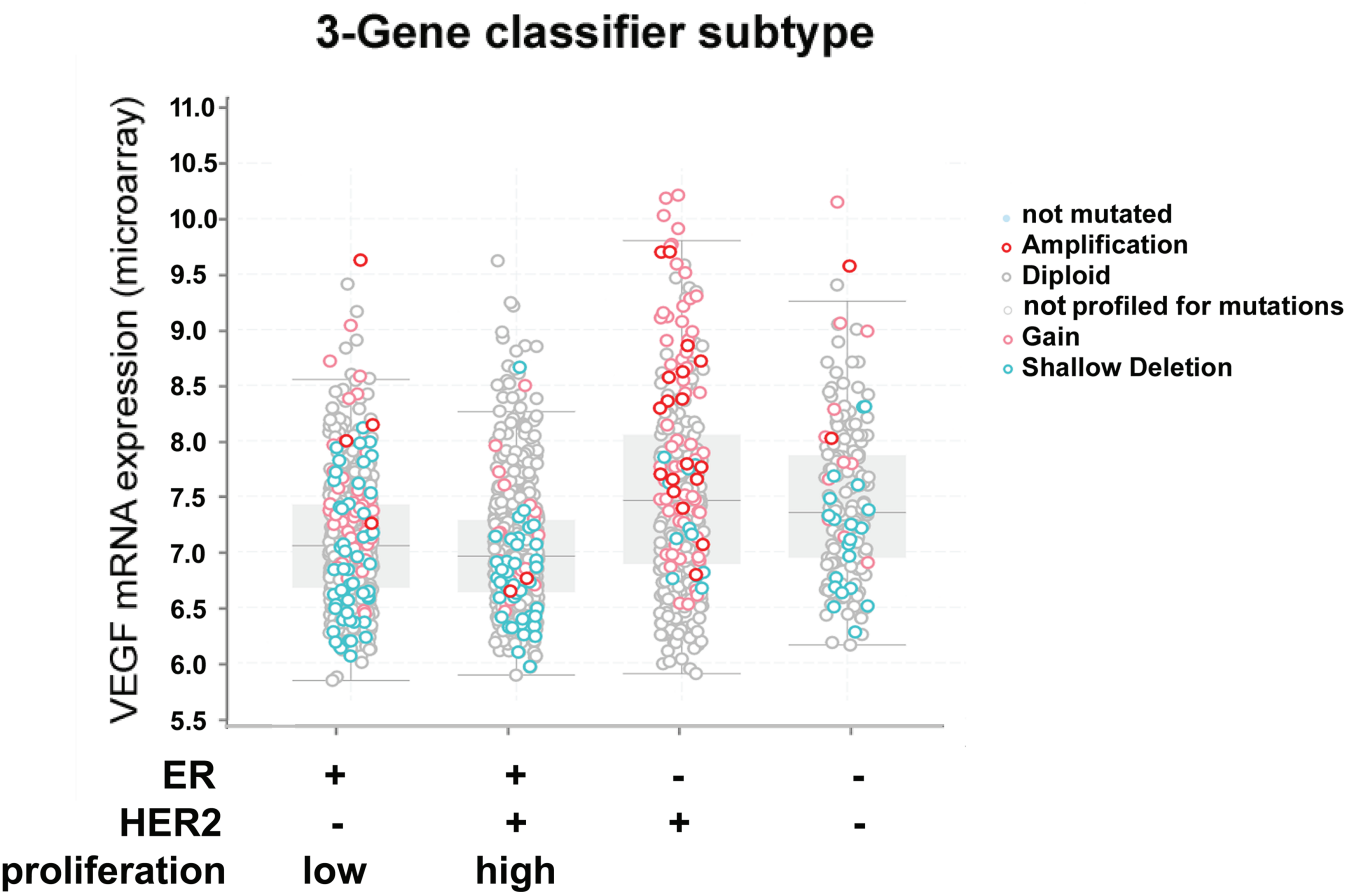
mRNA expression of vascular endothelial growth factor A (VEGFA) and genomic alterations in breast cancer specimens from the METABRIC study [31] in relation to BC receptor status. Data were obtained and analyzed using cBioportal [37]

As the TNBC cell line secreted the highest amounts of VEGF, we investigated whether this correlates with the results of our clinical samples. For this, we correlated the ER- and VEGF-scores by Spearman’s correlation analysis. There was indeed a significant negative correlation, but the correlation factor was low (ρ = −0.13, *p* = 0.047). To obtain further evidence for this observation and to correlate VEGF-mRNA abundance with the expression of key signaling molecules, we analyzed gene expression data from the METABRIC study [31] and the Cell Line Encyclopedia [32]. In METABRIC, VEGFA mRNA abundance was also highest in triple-negative tumors, associated with a high degree of gene amplification. Additionally, HER2/neu-positive tumors also exhibited high VEGFA mRNA expression (Table 2, Fig. 3). We also included IL1B and HIF1A, which encode important VEGFA-regulating signaling molecules, in this analysis (Table 2). IL1B expression was significantly higher in ER-, HER2/neu-negative cases, including TNBC cases. HIF1A was highly expressed in ER-, PR-negative, and TNBC cases. Pearson’s correlation factors were −0.18 (*p* < 0.01) for HIF1A to VEGFA, 0.03 for IL1B to VEGFA, and 0.21 (*p* < 0.01) for HIF1A to IL1B.

**Table 2:** Comparison of gene expression data from the METABRIC study according to receptor expression

BC Subtype	Expression Levels in METABRIC Study (Log-Scale)
	Mean ± Standard Error
	**One Way ANOVA *p* < 0.01
	VEGFA
ER−/+	7.58 ± 0.04/7.07 ± 0.02**
PR−/+	7.36 ± 0.03/7.04 ± 0.02**
HER2−/+	7.16 ± 0.02/7.41 ± 0.04**
TNBC−/+	7.11± 0.02/7.60± 0.05**
	IL1B
ER−/+	6.68 ± 0.03/6.54 ± 0.02**
PR−/+	6.59 ± 0.02/6.56 ± 0.02
HER2−/+	6.60 ± 0.02/6.37 ± 0.04**
TNBC−/+	6.53 ± 0.02/6.81 ± 0.04**
	HIF1A
ER−/+	7.00 ± 0.03/6.57 ± 0.02**
PR−/+	6.79 ± 0.02/6.56 ± 0.02**
HER2−/+	6.62 ± 0.02/6.98 ± 0.04
TNBC−/+	6.61± 0.02/6.97 ± 0.03**

Note: Mean of normalized mRNA expression (log-scale) of Interleukin-1β (IL1B), vascular endothelial growth factor A (VEGFA), and hypoxia-induced factor A (HIFA), in breast cancer subtypes of the METABRIC cohort was calculated, and significance was determined by one-way ANOVA test. **p < 0.01.

The data from the Cell Line Encyclopedia [32] demonstrated varying amounts of VEGFA-mRNA in TNBC cell lines (Table S1). On average, VEGFA was highest in HER2- followed by TNBC- and ER-positive cell lines. Only the difference between ER-positive and TNBC cell lines was significant (*p* = 0.016, ANOVA, Games-Howell post hoc test). HIF1A correlated with VEGFA (r = 0.274, *p* = 0.052) and IL1B (r = 0.339, *p* = 0.015), whereas IL1B correlated highly with VEGFA (r = 0.518, *p* < 0.001). In cluster analysis, using these three genes, TNBC cell lines also did not cluster together (Fig. S2).

### 3.3 IL-1*β* Activates NF-*κ*B and MAP-Kinase Signaling in Breast Cancer Cell Lines

**Figure 4:**
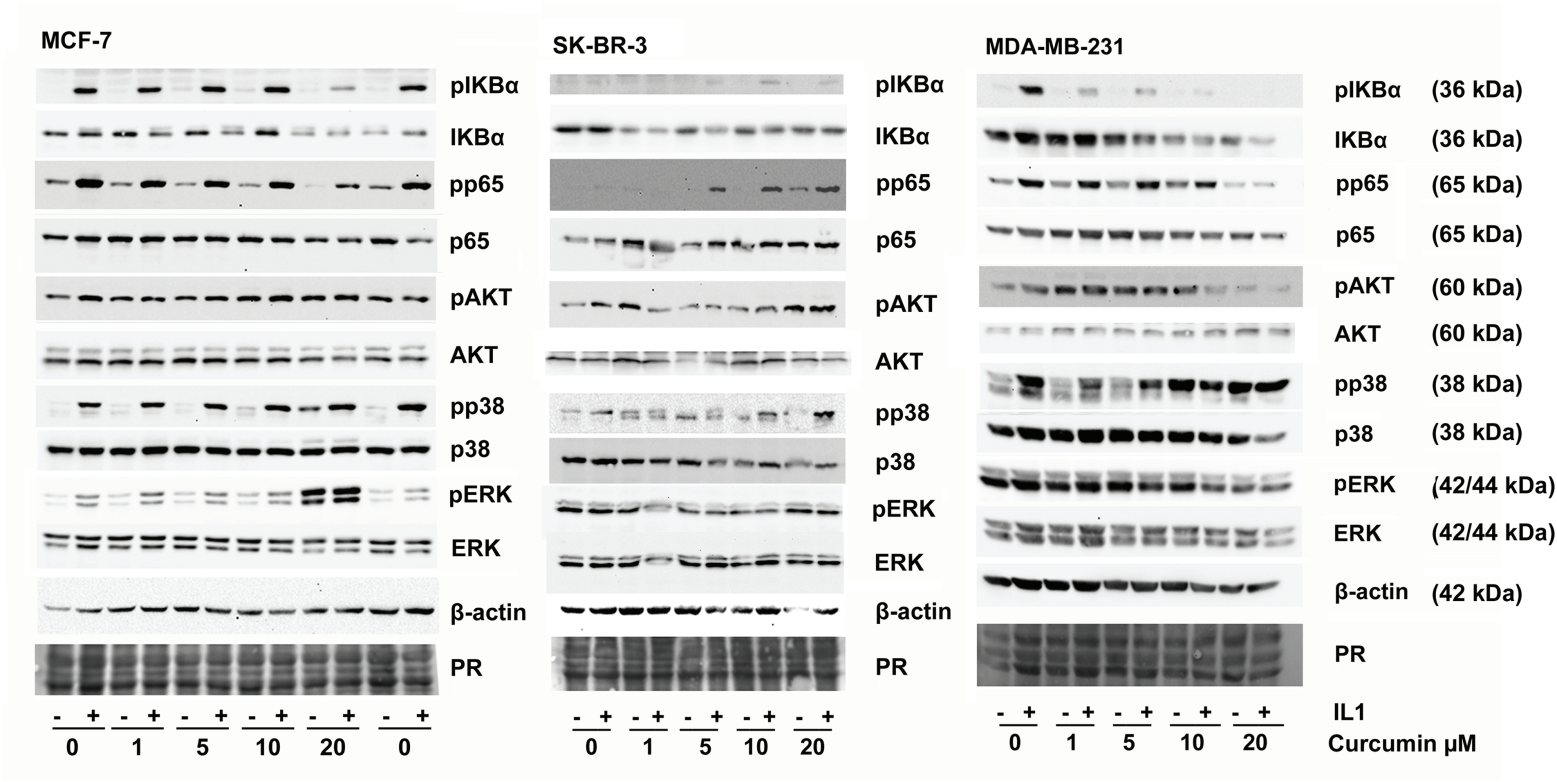
Phosphorylation of signaling proteins in response to curcumin and IL-1β was analyzed by Western blotting. Cells were pre-incubated with DMSO (solvent) or curcumin for 2 h before IL-1β (10 ng/mL) was added for 10 min. Cells were lysed, and the total protein was subjected to Western blot analysis. One representative result of three replicates is shown. PR: Protein stain

We then tested whether IL-1β was able to activate the inflammatory NF-κB transcription factor and thereby cause an increase in VEGF secretion in MCF-7, SK-BR-3, and MDA-MB-231 cell lines. Nuclear factor of kappa light polypeptide gene enhancer in B-cells (IκBα) inhibits NF-κB when unphosphorylated. Upon phosphorylation, it is degraded, and NF-κB is released. p65, also known as (RELA), is part of the NF-κB complex and is also activated by phosphorylation [44]. Thus, we estimated NF-κB activity by determining the amount of phosphorylated and non-phosphorylated IκBα and p65 by Western blotting. By these means, strong activation of NF-κB by IL-1β could be demonstrated in MCF-7, MDA-MB-231, but only moderately in SK-BR-3 (Fig. 4). We also investigated the activation of the mitogen-activated protein kinase (MAPK) pathway represented by the extracellular signal-regulated kinase (ERK), the stress-responsive p38-MAPK, as well as protein kinase B (AKT). ERK was only phosphorylated in response to IL-1β in MCF-7. Phospho-p38-MAPK was strongly increased in MCF-7, MDA-MD-231, but only modestly in SK-BR-3, whereas AKT was not affected in the cell lines.

### 3.4 Curcumin Affects IL-1-Mediated Activation of NF-*κ*B, MAP-Kinases, and VEGF Secretion

**Figure 5:**
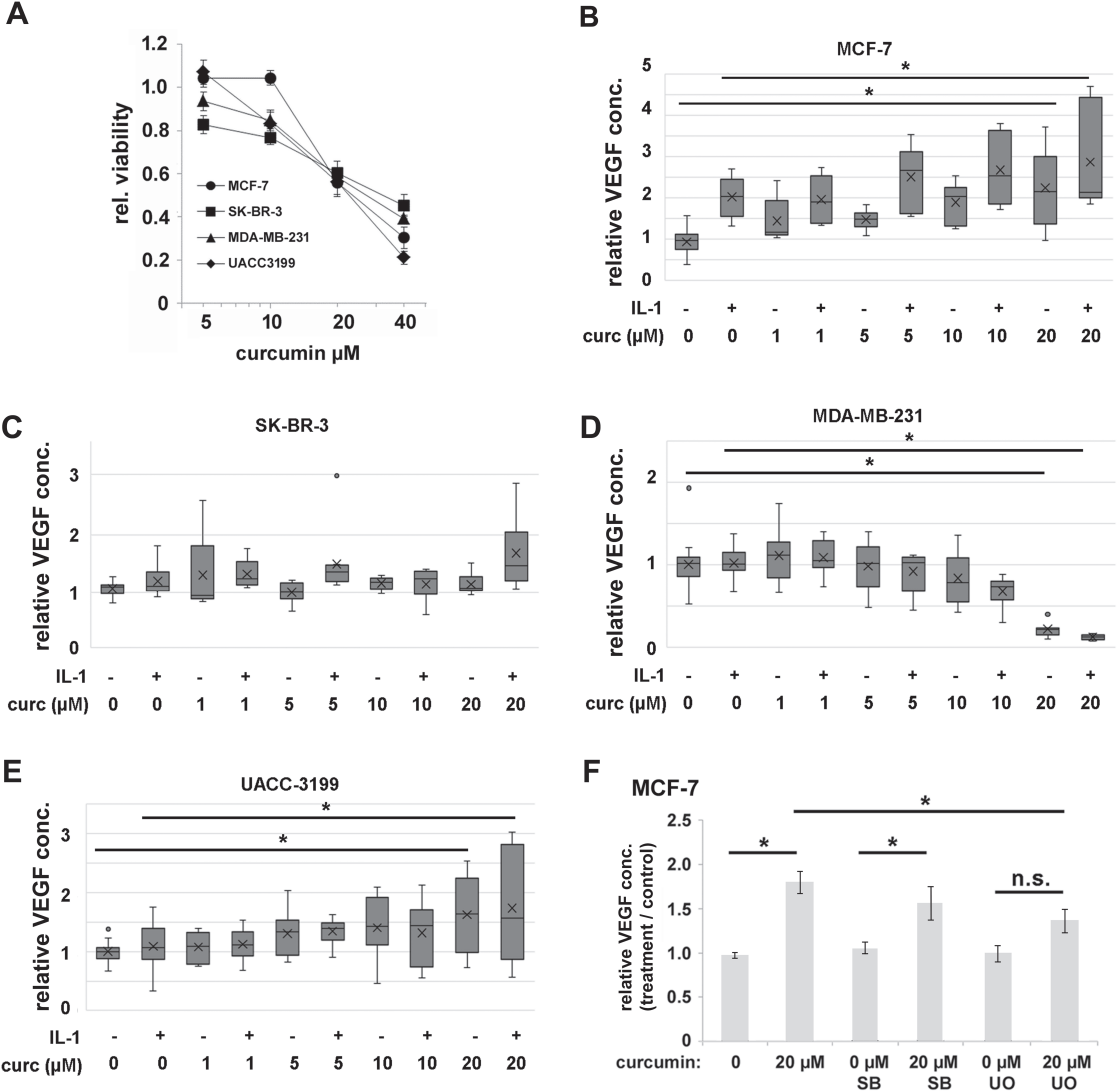
(**A**). Cell viability of cells treated with curcumin for 24 h. Viability was determined by the resazurin assay. Data were averaged from three independent experiments with six replicas each, and the standard deviation is shown. (**B)**. VEGF concentrations in cell culture supernatant upon 24 h exposure to IL-1β, curcumin, and combinations of both were determined by ELISA for (**B**) MCF-7, (**C**) SK-BR-3, (**D**) MDA-MB-231, and (**E**) UACC-3199. Data are averaged from three independent experiments with two replicas each, and median and quartiles are shown. (**F)**. Effects of inhibition of MAP-kinases on curcumin-induced VEGF secretion in MCF-7. SB: SB203580 (p38-MAPK-inhibitor); UO: UO126 (ERK-inhibitor). Data are expressed relative to solvent-treated cells. Data averaged from three independent experiments with three replicas each, and the standard error is shown. *p < 0.05, n.s.: not significant (ANOVA).

In parallel, we investigated whether curcumin might interfere with IL-1β-mediated VEGF secretion as previously seen in the chondrosarcoma cell lines [19]. Consistent with that study, our initial goal was to find a curcumin concentration that inhibited NF-κB signaling but caused only moderate viability loss for the cells. Curcumin showed similar toxicity to the four cell lines, and a concentration of 20 µM resulted in about 40% viability loss in 24 h (Fig. 5A). MCF-7 turned out to be slightly more tolerant to curcumin than the other cell lines; nevertheless, the differences between the cell lines in this assay were not substantial (Fig. 5A). At higher concentrations (up to 40 µM), we found significant cell death, and the results became unreliable. Thus, we continued this study with curcumin concentrations up to 20 µM. Surprisingly, increasing concentrations of curcumin alone resulted in elevated extracellular VEGF in some cell lines (Fig. 5B–E). Especially MCF-7 cells showed this effect already starting at 1 µM, UACC-3199 showed a nearly linear increase of VEGF secretion in the same concentration range, but there was no change in SK-BR-3 cells. Only in MDA-MB-231 cells, VEGF secretion was strongly reduced by curcumin at 10 and 20 µM curcumin.

We then analyzed NF-κB and MAP-kinase activation (Fig. 4) after adding IL-1β was altered in the presence of increasing concentrations of curcumin. We observed an inhibition of IL-1β-induced NF-κB activation (phosphorylation of IκBα and p65) by curcumin for MCF-7 and MDA-MB-231. However, in SK-BR-3, phosphorylation of IκBα and p65 was increased at higher curcumin concentrations (Fig. 4). In MCF-7 and MDA-MB-231, curcumin alone increased p38 MAPK-phosphorylation at concentrations above 10 µM. In MCF-7, ERK was also phosphorylated in response to curcumin alone. In SK-BR-3, no curcumin effect on ERK was apparent, and MDA-MB-231 showed a moderate decrease in pERK. Again, no effect on AKT was observed.

To further investigate the function of ERK and p38-MAPK for the VEGF secretion, we applied specific inhibitors for p38-MAPK (SB203580 [45]) and ERK (U0126 [46]) and determined the secreted amounts of VEGF. The applied inhibitor concentrations were established in a previous study and were shown to have only a weak effect on cell viability, but significantly inhibited the target kinases [47]. U0126 showed a significant inhibitory effect (0.76-fold of curcumin-induced increase in VEGF, *p* = 0.01 ANOVA, Tamhane T2-post hoc test) on VEGF secretion into the medium but not SB203580 (0.94-fold, *p* = 0.93, Fig. 5F).

### 3.5 Effect of Hypoxia on VEGF Secretion

**Figure 6:**
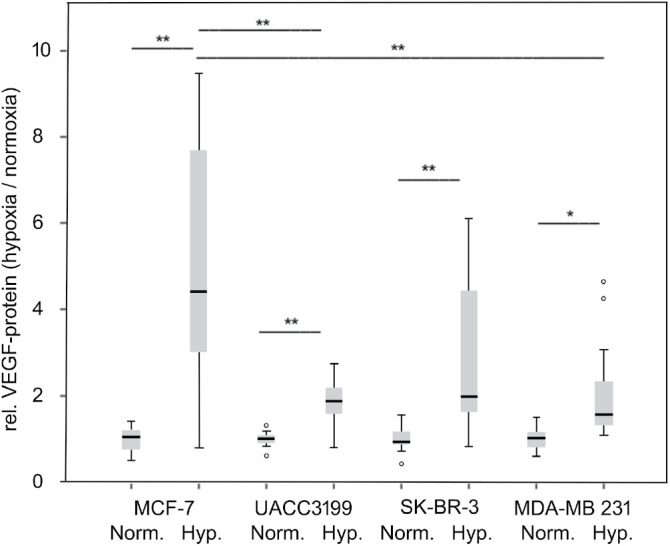
VEGF secretion in response to hypoxia. Cells were exposed to hypoxic conditions for 24 h, and VEGF was determined in the supernatant by ELISA. Norm.: normoxic, Hyp. hypoxic conditions. The average from three independent experiments, represented as box plots, and the median and quartiles are shown. *p < 0.05, **p < 0.01 (ANOVA, Tamhane T2 post hoc analysis), °: outliers

We next investigated whether VEGF secretion upon hypoxia also differed among the cell lines. However, in these experiments, all cell lines were able to increase VEGF in response to hypoxia (Fig. 6), though to varying degrees, with MDA-MB-231 displaying the lowest relative increase. In parallel, we analyzed the HIF1A mRNA in this experiment and observed a significant decrease in HIF1A mRNA under hypoxia in all cell lines tested after 24 h of hypoxia (Fig. S3).

## 4 Discussion

VEGF is a key regulator of tumor vascularization and a prognostic marker [46,48] in primary breast cancer, especially the TNBC subtype [49,50]. Anti-angiogenic treatment in combination with chemotherapy of TNBC has therefore attracted considerable interest; however, the results were not completely conclusive [16,51,52]. Whereas in the E2100 trial, the anti-VEGF antibody bevacizumab combined with chemotherapy approximately doubled the time until relapse [51,53], but there was no significant impact on overall survival. This was also valid in the BEATRICE trial focusing on early TNBC patients [54]. An improved prediction of therapy success and the development of combination therapies are therefore recommended. Recently, a combination with checkpoint inhibitor directed and/or chemotherapy has been evaluated in phase II studies showing promising results [55,56].

VEGF expression is mainly controlled by two pathways. The response to hypoxia is mediated by HIF1α [17], while also inflammatory signals such as IL-1β [57] can stimulate VEGF-expression. Indeed, IL-1β increases the invasiveness of TNBC cells via NF-κB, and this cytokine is also regulated by NF-κB in a positive regulatory loop [58]. IL-1β is also known as a driver of breast cancer growth and is especially associated with bone metastasis [18,21].

To obtain further evidence for the importance of VEGF abundance in clinical samples, we determined VEGF by IHC in our retrospective cohort. However, the results did not indicate a significant correlation with relapse and overall survival. We suggest this lack of association to the small cohort size, especially concerning TNBC cases, and also to the method applied. IHC detected VEGF in the cytoplasm of tumor cells, and we suspected that this reflects the amount of VEGF in the tumor microenvironment. However, secreted VEGF was not detected with this technique. Consequently, the immunoscore might not reflect the bioactive amount of VEGF in the tissue, and IHC might be questionable for determining active VEGF in tumor samples. On the other hand, it should be considered that mRNA levels do not necessarily correlate well with the active protein amount. Also, the gene expression studies analyze tumor tissue, not only consisting of cancerous cells, which could distort the gene expression levels to an unknown extent. The association of VEGF-expression with TNBC and HER2/neu overexpressing tumors observed in large transcriptome studies was also not significant in our cohort, and we again attribute this to the low number of TNBC cases (*n* = 32, *p* = 0.18) present. The observed significantly lower VEGF score in cases that did not undergo radiotherapy is hard to explain. We assume that this is a result of the complex selection criteria of patients for radiotherapy at the time the cohort was established. Interestingly, a low VEGF score correlated significantly with ductal compared to lobular BC. This corresponds to earlier observations indicating that angiogenesis and VEGF expression are different in lobular BC [59], where VEGF has a reduced impact on vessel formation. Also, lobular BC has a different pattern of infiltrating immune cells, thus, presumably a different composition of inflammatory cytokines, which might correlate with VEGF-expression [60].

The MDA-MB-231 cell line, representing the aggressive TNBC subtype, secreted the highest VEGF amounts, and these were not further increased by IL-1β. This result is in line with VEGF expression in TNBC in the clinical samples from the METABRIC study [31]. VEGF, therefore, likely contributes to the unfavorable outcome of TNBC by improving the nutrient and oxygen supply for such fast-growing tumors. However, the highly DNA-methylated and therefore BRCA-1-deficient UACC-3199 [61,62] TNBC cell line secreted significantly less VEGF than all other cell lines. Data from the Cell Line Encyclopedia [32] also support the observation that VEGF expression varies within the TNBC cell lines, maybe reflecting the different subtypes of TNBC [12], mutational landscape, or VEGFA-gene amplification. All cell lines tested responded to hypoxia by a significant increase in VEGF in the cell culture supernatant. This shows that the HIF-1α pathway is intact in all cells, although the basal VEGF expression varied significantly. Further proof for this is the observed changes in HIF1A mRNA (Fig. S3). However, after 24 h of hypoxia, the mRNA was unexpectedly reduced in contrast to the VEGF protein. HIF-1α is regulated mainly post-transcriptionally by stabilization of the protein [17]. The observed mRNA reduction may reflect negative feedback regulation.

This investigation was based on the hypothesis that IL-1β activates VEGF biosynthesis and secretion in breast cancer via NF-κB signaling, a process that can potentially be inhibited by curcumin. Additionally, curcumin is toxic to several cancer cell lines. Thus, curcumin could be beneficial for breast cancer patients. Our data on curcumin toxicity are consistent with studies published earlier by Shao et al. [63], Liu et al. [64], or Prasad et al. [65]. However, we observed a differential response of VEGF to IL-1β as well as to curcumin in the cell models tested. Only the Luminal A cell line MCF-7 responded to IL-1β by significantly increasing VEGF in the medium (Fig. 1B) about 2.5-fold. Curcumin inhibited or even increased VEGF secretion and NF-κB signaling depending on the cell line. Nevertheless, MDA-MB-231 and MCF-7 were both responsive to IL-1β as shown by NF-κB activation. Curcumin, on the other hand, inhibited IL-1β-mediated NF-κB activation in MDA-MB-231 and MCF-7 as proposed. But only the MDA-MB-231 (TNBC) showed the expected decrease in VEGF, parallel to the inhibition of NF-κB. Consistent with our data for the triple negative MDA-MB-231 cell line, a decreased expression of VEGF by curcumin has been reported *in vitro* [64], as well as curcumin caused decreased angiogenesis in a xenotransplant model [29,66] based on the same cell line. The authors associated curcumin toxicity and its regulation of VEGF secretion with inhibition of NF-κB activity [64] or the Wnt/β-catenin pathway [65]. Our data on NF-κB inhibition by curcumin are consistent with this proposal. However, the second TNBC cell line in this study, the BRCA-mutated UACC-3199, behaved significantly differently from MDA-MB-231. It had low basal levels of VEGF in the cell culture supernatant, which were increased by curcumin. This cell line is characterized by a high degree of DNA-methylation, and we can only assume that this influences the activity of the VEGFA gene. Supporting this, other studies have also shown that curcumin can target promoter methylation of DLC1 [67], leading to growth inhibition.

The luminal A cell line MCF-7 responded to curcumin by increasing VEGF secretion. Notably, curcumin can interact with estrogen signaling [63] and was also able to restore tamoxifen sensitivity [68] in MCF-7-derived tamoxifen-resistant cells. In contrast, in T-47D, another ER-positive cell line, curcumin reduced medroxyprogesterone-induced VEGF secretion [30] (see below).

The HER2/neu overexpressing SK-BR-3 also displayed a different response pattern. It exhibited a higher IL-1β-mediated and IL-1β-independent NF-κB activation when curcumin was added. It can be speculated that this is caused by the high activity of the receptor tyrosine kinase HER2/neu. Such kinases are known targets of curcumin [69].

Thus, the molecular mechanisms inducing cell death and regulating VEGF secretion might therefore vary for each cell line and tumor. For BC treatment with anti-VEGF antibodies, an increased VEGF secretion in response to curcumin would potentially limit the efficacy of this intervention rather than enhance it. It seems, therefore, desirable to predict such a response. Based on our data, with only a limited number of cell lines, this does not currently seem feasible.

To understand the mechanism of curcumin-induced VEGF secretion in MCF-7 further, we investigated the signaling events using inhibitors of MAP-kinase signaling. These experiments indicated that ERK activation was at least partially responsible for curcumin-induced VEGF secretion, whereas p38-MAPK appeared not to be involved. ERK activation by curcumin has been observed in other cells, e.g., leukemic THP-1. In these cells, an activation of the ERK/JNK/jun pathway was pro-apoptotic [70]. In contrast, in neural progenitor cells, low concentrations of curcumin activated ERK and p38-MAPK, presumably as a stress-response, whereas higher concentrations had cytotoxic effects [71,72]. A potential molecular mechanism discussed in the literature is the anti-oxidative capacity of curcumin, which counteracts stress-related reactive oxygen species [73]. In other cell lines, however, curcumin caused an inhibition of ERK/MAPK activity and thereby proliferation, such as in gastric carcinoma cells [74]. In lung cancer A549 cells, curcumin decreased proliferation mediated by ERK-activation of autophagy [75]. A similar mechanism was found for acute lymphoblastic leukemia cells [76]. In several tumors, curcumin interferes with receptor tyrosine kinases, resulting in inhibition of MAPK-signaling [69], thereby reducing cancer cell proliferation. Curcumin can also increase phosphorylation of MAP-kinases by inactivating protein phosphatases 2A and −5 [77], thereby leading to cell death in sarcoma and colon adenocarcinoma. Altogether, the effects of curcumin on ERK-signaling seem complex, and inhibition as well as activation through various pathways were observed. An involvement of ERK activation in VEGF production is known for M-CSF-induced VEGF production in monocytes [78]. Also, another small molecule, the oncometabolite succinate, was found to induce angiogenesis via ERK-mediated VEGF production [79].

Although p38-MAPK is a well-known regulator of VEGF expression [80], it seems to be less important for the curcumin-induced VEGF secretion in MCF-7 cells. p38-MAPK is a major stress-responsive kinase, regulating not only the expression of VEGF but also of HIF1α in solid cancers. *Vice versa*, VEGF activates ERK and p38-MAPK via the VEGF-receptor [81]. p38-MAPK was clearly activated in our experiments by IL-1β and also with higher curcumin concentrations; however, the extent was cell line specific, and only MCF-7 showed an IL-1β-induced VEGF secretion.

Nevertheless, further studies are needed to understand the different responses of the breast cancer cell lines and the underlying mechanisms in detail. It has been reported that curcumin inhibits progesterone-induced VEGF biosynthesis in another luminal-A cell line, T-47D. T-47D differs from MCF-7, e.g., by higher PgR expression, the presence of a p53 mutation, and morphology [82]. However, it is not clear whether p53- or PgR-status and associated differences in gene expression and activity of signaling pathways might cause this apparent different behavior towards curcumin. Differences might also lie in the increased activity of STAT3 in TNBC and ER-negative breast cancer [83,84] or different activity of the WNT-pathway, both known targets for curcumin [85]. Besides these molecules, cell lines may display variant activities of several other signaling pathways, such as NF-κB, p38-MAPK, and ERK, or the expression of the IL-1 receptors. This study does not provide sufficient data to fully elucidate the molecular mechanism for the differential responses of the cell lines in detail and can only be regarded as a starting point for further comparative investigations.

A major drawback for the therapeutic application of curcumin is its insufficient oral bioavailability; thus, curcumin serum concentration might not exceed 1 µM after oral administration of even large amounts [86,87]. At this concentration, we already observed effects on VEGF-secretion in two of the four cell lines investigated, but no significant cell death under our laboratory conditions. Several strategies are currently followed to increase the concentration in treated tumors. There are invasive options to increase the circulating or intra-tumor concentration, such as intravenous, intraperitoneal, or even intratumoral injections or transdermal application [88]. Such methods are frequently combined with the use of novel formulations of curcumin loaded on nanoparticles of varying chemistry [89–91]. These formulations can be based on liposomes, polymeric nanoparticles, carbon-dots, or nanoemulsions, to name a few [92–94]. Also, curcumin can be delivered to its target cells by, e.g., EGF-conjugated phospholipid particles [95]. Such formulations also have the potential to achieve significantly higher curcumin serum levels via oral administration [92–94]. Although such formulations are still in testing, we assume that applying curcumin in cell cultures at significantly higher concentrations than 1 µM can yield meaningful data. Our data suggest that TNBC patients might benefit from curcumin-induced cell death and reduced VEGF secretion. In contrast, in luminal A tumors, represented by MCF-7, insufficient concentrations of curcumin might have adverse effects due to possibly increased tumor vascularization. However, it is not clear to what extent such cell-based studies predict *in vivo* effects. To answer such questions, additional clinical or animal studies are needed [96–98].

The most intriguing characteristic of curcumin is that it is notable for a wide range of effects, ranging from inhibition of proliferation and inflammation to the modulation of cholesterol homeostasis. This seems quite intriguing, but in fact, several of these data could be misleading. Curcumin is rarely used as a pure substance, and it is also differentially metabolized in cell cultures. Indeed, some researchers consider curcumin, therefore a PAIN (panassays interfering compound) and an IMP (invalid metabolic panaceas) [99]. Often, curcumin effects are observed near toxic concentrations. We here identified curcumin effects at intermediate concentrations where only limited cell death occurred. Although we could link the increased VEGF secretion in the MCF-7 cell line to ERK-phosphorylation, further studies are needed to understand this effect at the molecular level.

To obtain further information on the connection of VEGF expression with IL-1β or HIF1α in breast cancer, we further investigated mRNA datasets regardless of BC subtype. Although protein abundance and modifications are more important for these pathways, mRNA expression often follows function, as activation of signaling proteins regularly induces a higher turnover. We therefore reasoned that analyzing mRNA abundance might lead to meaningful results. Indeed, in the METABRIC study, TNBC had the highest *VEGFA*- as well as *IL1B* mRNA levels. However, there was no relevant correlation between *VEGFA* and *IL1B* RNA. Unlike simple comparisons of averaged expression values, a correlation analysis investigates paired patient data and is therefore more informative. This result holds against a direct regulatory association between *VEGFA* and *IL1B* in tumors. In the breast cancer cell lines database, however, *VEGFA* correlated with *IL1B*, and this suggests that *IL1B* is more important for *VEGFA* under normoxic *in vitro* conditions than in tumors, where cells are embedded in a supporting microenvironment and subject to hypoxia. Nevertheless, the correlation factor was 0.52, indicating that this correlation is far from being perfect. Our finding that only MCF-7 responded to IL-1β by VEGF production clearly supports this observation. A multifactorial analysis, including the mutational landscape and gene amplifications in *VEGFA* or *IL1B*, may help identify further factors influencing IL-1β induction of VEGF biosynthesis. Altogether, our analysis suggests that *VEGFA* regulation via *IL1B* and, consequently, NF-κB is more important *in vitro* than in patients’ tumors. On the other hand, the correlation between *IL1B* and *HIF1A*, present in both datasets, demonstrated a cross-talk between the two pathways.

## 5 Limitations of this Study

This study relies on experiments performed with only one cell line representing each of the major BC subtypes. To what extent our observations translate to patients is therefore not clear. Further studies on patient-derived organoids or xenografts are needed to improve the generalization of our observations. Furthermore, curcumin is metabolized into several derivatives [100] that might be responsible for different effects *in vitro* and *in vivo*. Such metabolites should be determined in the cell culture models. It should also be mentioned that the statistical power of some experiments performed three times with 2–6 replicas each might be too small to detect small differences with sufficient sensitivity. Also, the statistical power of our retrospective cohort is low compared to the METABRIC study. This, and the discussed differences in methodologies limit the comparability of the two datasets.

## 6 Conclusions

In conclusion, we observed substantial variation in response to IL-1β and curcumin across a small panel of breast cancer cell lines. Curcumin had opposing effects on VEGF secretion, despite causing cell death in all four cell lines within a similar concentration range. Gene expression data from tumors suggest that VEGFA regulation is complex and involves an interplay of at least two major pathways, NF-κB and HIF. Together, these observations suggest that the outcome of a therapy approach based on curcumin as well as on α-IL-1β antibodies will vary within the breast cancer subtypes.

## Supplementary Materials



## Data Availability

Further data that support the findings of this study are available from the Corresponding Author, [Norbert Nass], upon reasonable request.

## Supplementary Materials

The supplementary material is available online at https://www.techscience.com/doi/10.32604/or.2025.072793/s1. Fig. S1: Relative vascular endothelial growth factor (VEGF) in growth medium, 24 h after addition of interleukin-1β (IL-1β). Table S1: Vascular endothelial growth factor A (*VEGFA*) mRNA expression in breast cancer cell lines. Fig. S2: Breast cancer cell lines clustered according to gene expression of VEGFA, HIF1A and IL1B Fig. S3: *HIF1A* mRNA abundance in breast cancer cell lines.
