# Supplementary material for: Interplay of Interleukin-1β and Curcumin on VEGF Expression in Breast Cancer Cells
Source: Oncol Res. 2026 Feb 24;34(3):13. doi: 10.32604/or.2025.072793 (PMC12963662; doi:10.32604/or.2025.072793)
Supplement: Supplementary file 1 [file OncolRes-34-72793-s001.docx]

Supplement Nass et al.


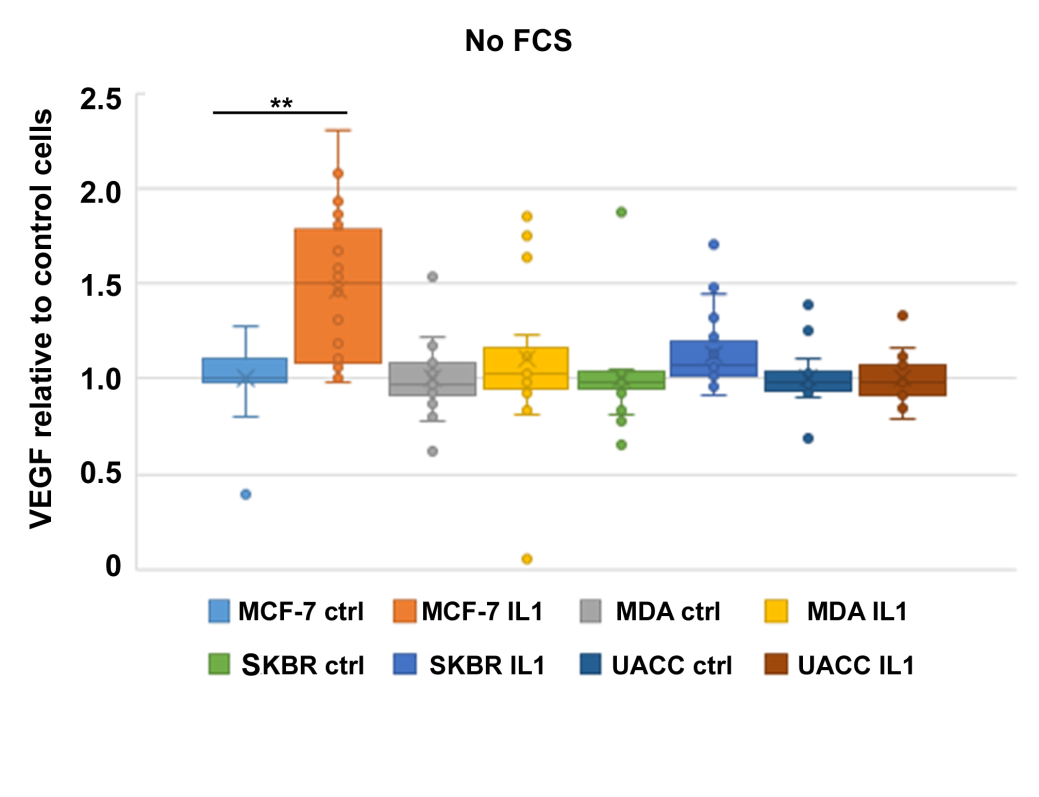


**Figure S1:** Relative vascular endothelial growth factor (VEGF) in growth medium, 24 h after addition of interleukin-1β (IL-1β). The experiment was performed in the same way as described in the main manuscript, except that cells were incubated in the absence of fetal calf serum (FCS) to exclude the influence of factors derived from FCS. VEGF concentrations in serum were normalized to the results from respective control cells (only solvent added). The experiment was done three times with 4 replicas each. Data are represented as box plots with median (−−−), average (x), quartiles and outliers (•) indicated. **: *p* < 0.01 (ANOVA, Games-Howell post-hoc test).

| Cell line | characteristics | VEGFA (microarray) | IL1B (microarray) | HIF1A (microarray) |
| --- | --- | --- | --- | --- |
| T47D | ER+ PR+ | 6.534335 | 4.643914 | 11.77209 |
| HCC38 | TNBC | 6.746489 | 4.652053 | 12.4127 |
| EFM19 | ER+ PR+ | 7.076975 | 4.811988 | 12.20096 |
| KPL1 | ER+ | 7.101066 | 4.501558 | 11.80305 |
| HCC1806 | TNBC | 7.119665 | 8.140837 | 12.07982 |
| DU4475 | TNBC | 7.273369 | 5.758141 | 11.72148 |
| MDAMB134VI | ER+ | 7.383802 | 4.630471 | 12.25971 |
| HCC1419 | ER+/- HER2 | 7.42446 | 4.878478 | 12.41276 |
| BT549 | TNBC | 7.450805 | 5.460277 | 12.23692 |
| BT20 | TNBC | 7.489089 | 4.511695 | 12.95014 |
| MDAMB361 | ER+ PR+/- HER2 | 7.591169 | 4.684841 | 12.48805 |
| ZR751 | ER+ PR+/- | 7.616905 | 4.604444 | 11.49642 |
| EFM192A | ER+ PR+ HER2 | 7.637167 | 4.463533 | 12.30608 |
| CAL120 | TNBC | 7.725108 | 4.488371 | 12.35259 |
| BT474 | ER+ PR+ HER2 | 7.726945 | 4.781922 | 12.69506 |
| SKBR3 | HER2 | 7.73523 | 4.6946 | 11.61709 |
| HCC1500 | ER+/- PR+/- | 7.780089 | 4.683147 | 10.99385 |
| MCF7 | ER+ PR+ | 7.803549 | 4.3317 | 12.21055 |
| HCC1428 | ER+ PR+ | 7.821575 | 4.760133 | 12.1455 |
| BT483 | ER+ PR+/- | 7.824183 | 4.594403 | 13.06269 |
| AU565 | HER2 | 7.845598 | 4.741511 | 12.29785 |
| CAL148 | TNBC | 8.034744 | 4.57983 | 12.7034 |
| HMC18 |  | 8.038872 | 4.995742 | 11.29744 |
| MDAMB453 | HER2 | 8.052762 | 4.671927 | 11.21194 |
| ZR7530 | ER+ HER2 | 8.058325 | 5.021093 | 12.18754 |
| HCC1599 | TNBC | 8.067534 | 4.791886 | 13.31688 |
| MDAMB415 | ER+ PR+/- | 8.192692 | 5.038912 | 12.17798 |
| MDAMB468 | TNBC | 8.22848 | 4.796695 | 12.28616 |
| UACC812 | ER+ PR+/- HER2 | 8.300713 | 4.449686 | 12.23655 |
| HS281T | ER+ | 8.301142 | 4.736417 | 13.46291 |
| CAMA1 | ER+ PR+/- | 8.334315 | 4.776614 | 10.97479 |
| JIMT1 | HER2 (resistent) | 8.408096 | 4.886627 | 12.14417 |
| HCC1187 | TNBC | 8.531285 | 4.981163 | 11.73525 |
| CAL51 | TNBC | 8.585019 | 4.530014 | 12.29671 |
| HCC70 | TNBC | 8.630083 | 5.378028 | 12.86996 |
| HCC1143 | TNBC | 8.633788 | 4.737955 | 11.92239 |
| HCC2218 | HER2 | 8.641628 | 5.082394 | 11.23434 |
| MDAMB436 | TNBC | 8.653163 | 11.52941 | 12.42655 |
| HCC202 | HER2 | 8.733556 | 4.684959 | 11.74798 |
| HCC1954 | HER2 | 8.762401 | 5.414187 | 12.91034 |
| UACC893 | HER2 | 8.781033 | 4.689204 | 12.26857 |
| HS739T |  | 8.864307 | 5.817382 | 13.08027 |
| EVSAT | ER- PR+/- HER2+/- | 8.923633 | 4.919028 | 11.72813 |
| HCC2157 | TNBC | 8.965314 | 4.864071 | 11.78287 |
| HS343T |  | 9.086584 | 7.580337 | 13.5776 |
| MDAMB231 | TNBC | 9.304904 | 5.060567 | 12.77957 |
| CAL851 | TNBC | 9.628472 | 7.995367 | 12.61203 |
| HCC1569 | HER2 | 9.736668 | 4.637525 | 11.24385 |
| HS742T |  | 10.5949 | 10.79913 | 13.24992 |
| HCC1395 | TNBC | 10.65287 | 5.332779 | 12.76141 |
| HS578T | TNBC | 11.33124 | 10.56532 | 13.09072 |

**Table S1. Vascular endothelial growth factor A (*VEGFA*) mRNA expression in breast cancer cell lines.** Microarray gene expression data from the cell line encyclopedia were obtained via cBioportal. Expression values were color-coded from low (green) to high (red) separately for each gene. Triple negative breast cancer (TNBC) cell clines are labelled in yellow; Cell lines analysed in this study are shown in blue. *VEGFA* expression is shown from low (green) to high (red). ER: estrogen receptor, PR: progesterone receptor; HER2: Human Epidermal Growth Factor Receptor 2, IL1B Interleukin-1β, HIF1A: Hypoxia-Induced Factor 1A


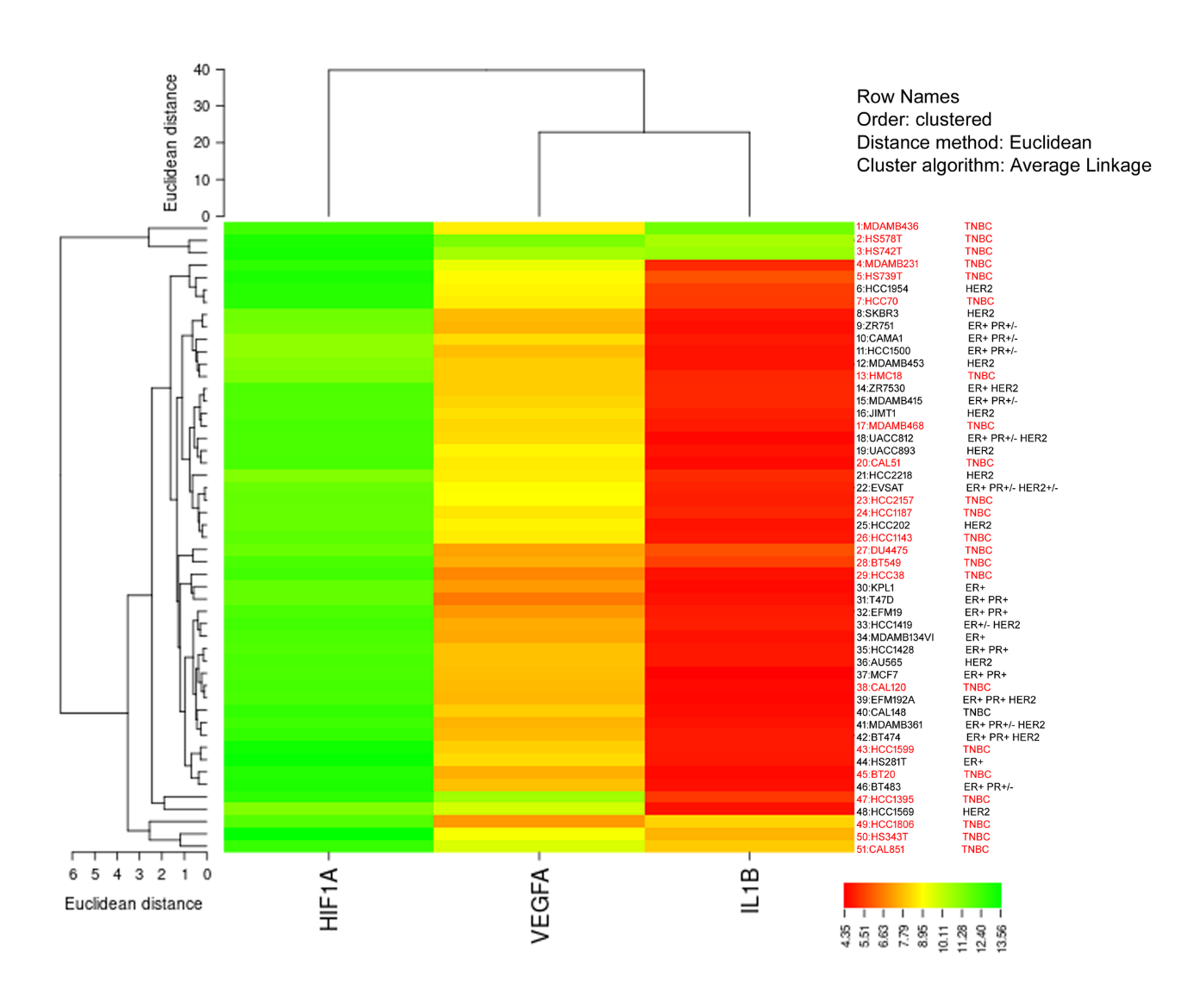


**Figure S2**. Breast cancer cell lines clustered according to gene expression of VEGFA, HIF1A and IL1B. Triple negative breast cancer (TNBC) cell lines are labelled in red. The data correspond to Table S1. Cluster analysis was done using the CimMiner website (https://discover.nci.nih.gov/cimminer/)

ER: Estrogen receptor, PR: Progesterone receptor; HER2: Human epidermal growth factor receptor 2, HIF1A: Hypoxia-induced factor 1A.


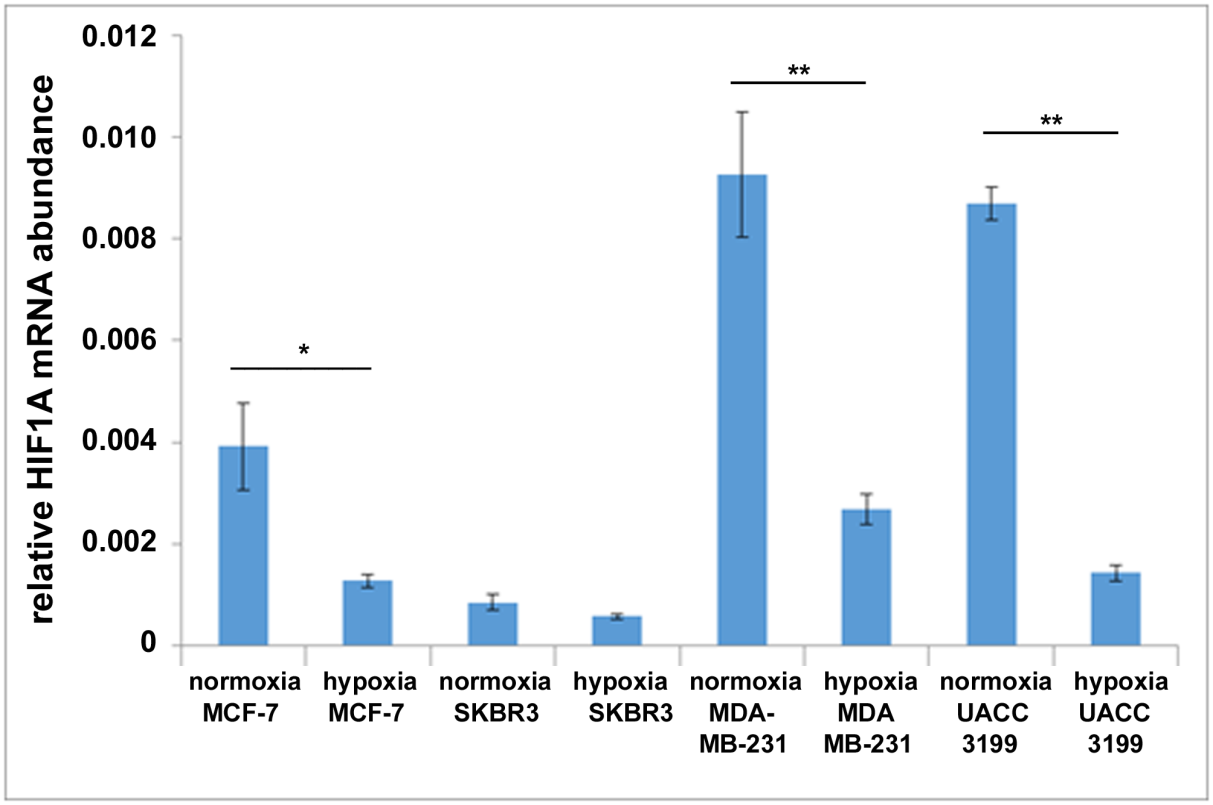


**Figure S3.** *HIF1A* mRNA abundance in breast cancer cell lines. Cells were exposed to normoxia/hypoxia for 24h and RNA extracted. cDNA was synthesized and analyzed by real-time polymerase chain reaction. *HIF1A* mRNA abundance was normalized to ribosomal protein L13 (*RPL13*) mRNA. *: *p* < 0.05; **: *p* < 0.01 (ANOVA, Bonferoni post-hoc test).
